# Editorial

**DOI:** 10.1093/sleepadvances/zpaa001

**Published:** 2020-03-12

**Authors:** Mary A Carskadon

**Affiliations:** Alpert Medical School, Brown University, Providence, RI, USA

Welcome to *Sleep Advances*, the new journal of the Sleep Research Society (SRS). It is my privilege and honor to serve as the inaugural editor-in-chief of this online-only, gold open access journal that will help propel the field forward by publishing excellent papers in sleep and circadian research. Our vision is to disseminate timely developments and advances that reflect the diversity of sleep and circadian science and to offer our authors quick, objective, and straightforward peer review.

Our mission is to build toward this goal with a strong and effective editorial board and peer reviewers. We plan for reviews that are fundamentally fair and reasonably fast. We will offer our authors a process for pre-submission inquiry with rapid turnaround. We will also provide a mechanism for excellent submissions that do not meet *SLEEP*’s high standard for acceptance to cascade to the *SLEEP Advances* review process so that this science can find a publication home in an SRS journal.

We will focus our content across the lifespan and across species, methodologies, and ideas. We welcome science that explores novel concepts and approaches, including basic biology, neuroscience, and behavior. Papers that introduce the field to new measures or methods of analysis will also be welcome. We are eager to publish excellent translational research, though hope that the field’s other clinically oriented journals will continue to publish papers that describe and report practice parameters and clinical guidelines.

Accomplishing our goals will depend on the active engagement of members of the sleep and circadian field from around the world that include scientists in North America, Latin America, Asia, Europe, Australia, Africa, and the Middle East. We seek both established and emerging leadership for our editorial team … first, second, and third generations of our scientists … who are ready to help launch and sustain the journal.

We envision supporting our authors by providing swift feedback on submissions along with special opportunities to present and highlight their science, including rapid publication and release free to the public, promotion of noteworthy articles to the media, and highlighting individual papers with “cover” art. We will support our authors to manifest transparency and reproducibility of their findings by encouraging them to provide full data sets and analytic code. Because of our stature as on-line, digital journal, we hope our authors will also take advantage of the format to submit videos, three-dimensional graphics, and other content that can enhance the visibility and accessibility of their work.

Again, I am thrilled to undertake leadership of *SLEEP Advances*, I am eager to work with the SRS leadership to guide the journal, and I am ready to welcome manuscripts from the best our field has to offer.

I thank John Noel, SRS Executive Director, Ron Szymusiak, EIC of *SLEEP*, and many individuals at Oxford University Press, including Christina Snyder, for their effort and encouragement in making the SRS vision of this journal a (virtual) reality.

